# Tetra­kis(benzyl­amino)­phospho­nium chloride

**DOI:** 10.1107/S1600536810054401

**Published:** 2011-01-08

**Authors:** Khodayar Gholivand, Hossein Mostaanzadeh, Sedigheh Farshadian

**Affiliations:** a Department of Chemistry, Tarbiat Modares University, Tehran, 14115/175, Iran

## Abstract

The title salt, [P(NHCH_2_C_6_H_5_)_4_]^+^·Cl^−^, crystallizes with the P atom and Cl^−^ anion lying on a twofold rotation axis. The P atom has a slightly distorted tetra­hedral geometry with two classes of N—P—N angles [101.15 (10) and 100.55 (11)° and 113.07 (9) and 114.83 (8)°] and the environments of *sp* ^2^-hybridized N atoms are essentially planar (sum of angles = 359.9 and 360.1°). In the crystal, the phospho­nium ion inter­acts with each neighboring chloride ion *via* two approximately equal N—H⋯Cl inter­actions, forming parallel chains along the *c* axis.

## Related literature

For background information on phospho­nium salts, see: Hart & Sisler (1964 ▶); Levason *et al.* (2006 ▶); Schiemenz *et al.* (2003 ▶). For related structures, see: Bickley *et al.* (2004 ▶); Horstmann & Schnick (1996 ▶)
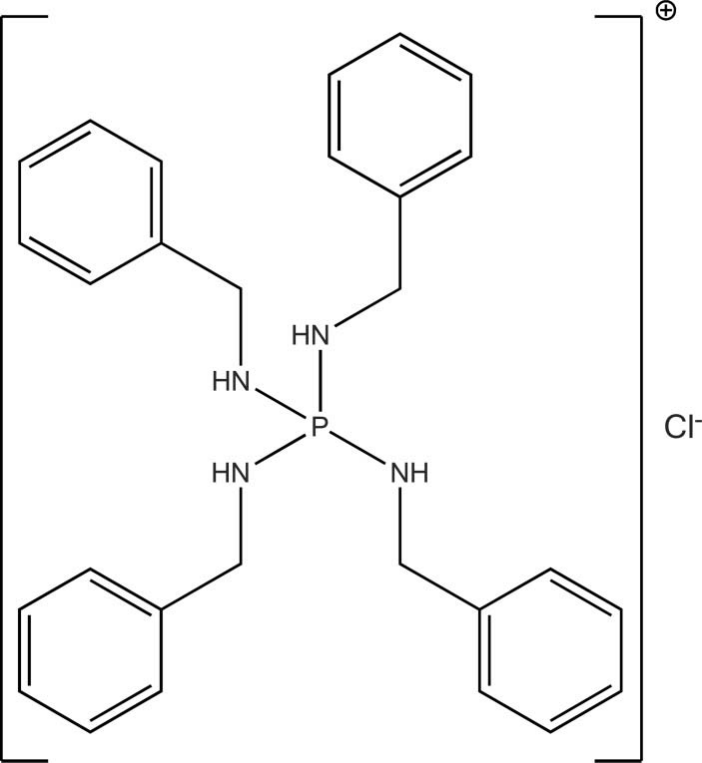


## Experimental

### 

#### Crystal data

C_28_H_32_N_4_P^+^·Cl^−^
*M* *_r_* = 491.00
Orthorhombic, 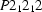

*a* = 11.359 (3) Å
*b* = 14.258 (4) Å
*c* = 7.923 (3) Å
*V* = 1283.1 (6) Å^3^
*Z* = 2
Mo *K*α radiation
μ = 0.24 mm^−1^
*T* = 193 K
0.50 × 0.40 × 0.25 mm

#### Data collection

Rebuilt Syntex P2_1_/Siemens P3 four-circle diffractometer
6760 measured reflections
3122 independent reflections
2677 reflections with *I* > 2σ(*I*)
*R*_int_ = 0.024
2 standard reflections every 98 reflections  intensity decay: 2%

#### Refinement

*R*[*F* ^2^ > 2σ(*F* ^2^)] = 0.036
*wR*(*F* ^2^) = 0.094
*S* = 1.01
3122 reflections
155 parameters
H-atom parameters constrained
Δρ_max_ = 0.35 e Å^−3^
Δρ_min_ = −0.33 e Å^−3^
Absolute structure: Flack (1983 ▶), 1321 Friedel pairs
Flack parameter: 0.08 (8)

### 

Data collection: *P3/PC* (Siemens, 1989 ▶); cell refinement: *P3/PC*; data reduction: *P3/PC*; program(s) used to solve structure: *SHELXTL* (Sheldrick, 2008 ▶); program(s) used to refine structure: *SHELXTL*; molecular graphics: *SHELXTL* and *Mercury* (Macrae *et al.*, 2008 ▶); software used to prepare material for publication: *SHELXTL*.

## Supplementary Material

Crystal structure: contains datablocks I, global. DOI: 10.1107/S1600536810054401/nk2077sup1.cif

Structure factors: contains datablocks I. DOI: 10.1107/S1600536810054401/nk2077Isup2.hkl

Additional supplementary materials:  crystallographic information; 3D view; checkCIF report

## Figures and Tables

**Table 1:** Hydrogen-bond geometry (Å, °)

*D*—H⋯*A*	*D*—H	H⋯*A*	*D*⋯*A*	*D*—H⋯*A*
N1—H1*C*⋯Cl1^i^	0.86	2.35	3.1848 (17)	163
N2—H2*A*⋯Cl1	0.86	2.35	3.1855 (18)	165

Symmetry code: (i) 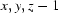.

**Fig. 1.**
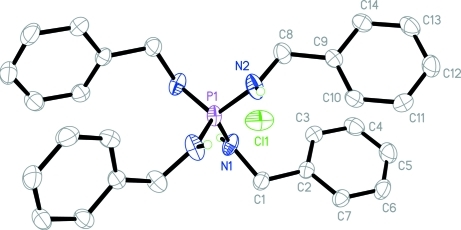
The structure of the title compound with 50% displacement ellipsoids and C-bound H atoms omitted. Unlabeled atoms are related to labeled atoms by a two-fold rotation.

**Fig. 2.**
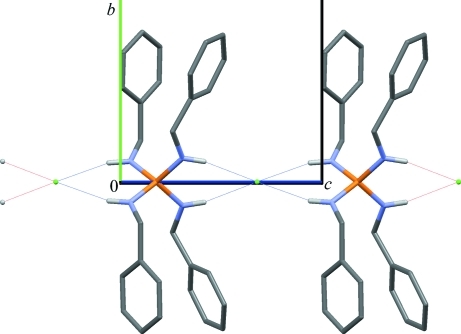
An a-axis projection showing hydrogen bonding dotted lines. The long b axis is truncated.

## supplementary crystallographic information

### Comment

Phosphonium salts, [PR4]^+^, are widely used as large cations to stabilize a variety of anionic species and to phase-transfer anions into low polarity organic media (Levason *et al.*, 2006). A few examples of tetraamino phosphonium salts exist in the literature (Hart & Sisler, 1964) and some of them are used as catalysis for preparing fluorine-containing compounds by a halogen/fluorine exchange reaction (Schiemenz *et al.*, 2003). Also, the crystal structure of P(NH_2_)_4_Cl (Horstmann & Schnick, 1996) and [P(NHPh)_4_]Cl (Bickley *et al.*, 2004) have been reported. In an effort to further investigation into these types of compounds, the crystal structure of [P(NHCH_2_C_6_H_5_)_4_]^+^.Cl^-^ is presented.

The title salt, [P(NHCH_2_C_6_H_5_)_4_]^+^.Cl^-^, crystallizes with the P atom and Cl^-^ anion lying on a two-fold rotation axis in the space group *P*2_1_2_1_2. The supramolecular structure of compound exhibits a polymeric chain of alternating phosphonium and chloride ions in the solid state (Fig. 2) with P···P distances of 7.923 Å. The phosphonium ion interacts with each neighboring chloride ion *via* two equal N—H···Cl interactions and six-membered rings around each phosphorus atom are formed. The centroid–centroid distance between adjacent phenyl rings is 4.009 Å, indicating no strong π–π stacking interactions exist in compound.

The four P–N bonds are of almost equal lengths 1.6166 (14) and 1.6184 (14) Å and are similar to those found in [P(NHPh)_4_]Cl (Bickley *et al.*, 2004) and P(NH_2_)_4_Cl (Horstmann & Schnick, 1996). These P–N bonds are shorter than the typical P–N single bond length (1.77 Å) . As observed in [P(NHPh)_4_]Cl, there are two classes of N–P–N angles resulting in a distorted tetrahedral environment for P1. More acute angles of 101.15 (10) and 100.55° are observed within the hydrogen bonding chelates of the N1–P1–N1^i^and N2–P1–N2^i^ (i: -*x*, -*y*, *z*) units respectively, whereas the remaining four N–P–N angles are 113.07 (9) and 114.83 (8)°. Although the main cause for the distortion from the ideal tetrahedral geometry is unclear, it seems to be partly controlled by hydrogen bonding. The sum of angles around nitrogen atoms are exactly 360° and these atoms are *sp*^2^ hybridized.

### Experimental

All the reagents and solvents were used as obtained without further purification. Phosphorus pentachloride (2 mmol) was added drop wise with constant stirring to a toluene solution (30 ml) of benzylamine (8 mmol) at 0°C. After an hour stirring, the reaction mixture was refluxed for 3 h. The solid formed during the reaction was filtered and then washed with distilled water, toluene, chloroform and dried. Single crystals suitable for single-crystal X-ray diffraction analysis were obtained from the mixture of MeOH and CH_3_CN solution.

### Refinement

The hydrogen atoms of NH groups were found in difference Fourier synthesis, the H(C) atom positions were calculated. All hydrogen atoms were refined in isotropic approximatiom in riding model with with the *U*_iso_(H) parameters equal to 1.2 *U*_eq_(*X*), where U(*X*) are the equivalent thermal parameters of the atoms to which corresponding H atoms are bonded. The absolute structure parameter (Flack, 1983) is refined based on 1321 Friedel pairs.

### Crystal data

**Table:**

C_28_H_32_N_4_P^+^·Cl^−^	*F*(000) = 520
*M**_r_* = 491.00	*D*_x_ = 1.271 Mg m^−^^3^
Orthorhombic, *P*2_1_2_1_2	Mo *K*α radiation, λ = 0.71073 Å
Hall symbol: P 2 2ab	Cell parameters from 24 reflections
*a* = 11.359 (3) Å	θ = 10–11°
*b* = 14.258 (4) Å	µ = 0.24 mm^−^^1^
*c* = 7.923 (3) Å	*T* = 193 K
*V* = 1283.1 (6) Å^3^	Plate, colourless
*Z* = 2	0.50 × 0.40 × 0.25 mm

### Data collection

**Table:**

Rebuilt Syntex P2_1_/Siemens P3 four-circle diffractometer	*R*_int_ = 0.024
Radiation source: fine-focus sealed tube	θ_max_ = 28.1°, θ_min_ = 2.3°
graphite	*h* = −15→15
θ/2θ scans	*k* = −18→18
6760 measured reflections	*l* = −10→10
3122 independent reflections	2 standard reflections every 98 reflections
2677 reflections with *I* > 2σ(*I*)	intensity decay: 2%

### Refinement

**Table:**

Refinement on *F*^2^	Secondary atom site location: difference Fourier map
Least-squares matrix: full	Hydrogen site location: difference Fourier map
*R*[*F*^2^ > 2σ(*F*^2^)] = 0.036	H-atom parameters constrained
*wR*(*F*^2^) = 0.094	*w* = 1/[σ^2^(*F*_o_^2^) + (0.0604*P*)^2^ + 0.1221*P*] where *P* = (*F*_o_^2^ + 2*F*_c_^2^)/3
*S* = 1.01	(Δ/σ)_max_ = 0.001
3122 reflections	Δρ_max_ = 0.35 e Å^−^^3^
155 parameters	Δρ_min_ = −0.33 e Å^−^^3^
0 restraints	Absolute structure: Flack (1983), 1321 Friedel pairs
Primary atom site location: structure-invariant direct methods	Flack parameter: 0.08 (8)

### Special details

**Table:**

Geometry. All e.s.d.'s (except the e.s.d. in the dihedral angle between two l.s. planes) are estimated using the full covariance matrix. The cell e.s.d.'s are taken into account individually in the estimation of e.s.d.'s in distances, angles and torsion angles; correlations between e.s.d.'s in cell parameters are only used when they are defined by crystal symmetry. An approximate (isotropic) treatment of cell e.s.d.'s is used for estimating e.s.d.'s involving l.s. planes.
Refinement. Refinement of *F*^2^ against ALL reflections. The weighted *R*-factor *wR* and goodness of fit *S* are based on *F*^2^, conventional *R*-factors *R* are based on *F*, with *F* set to zero for negative *F*^2^. The threshold expression of *F*^2^ > σ(*F*^2^) is used only for calculating *R*-factors(gt) *etc*. and is not relevant to the choice of reflections for refinement. *R*-factors based on *F*^2^ are statistically about twice as large as those based on *F*, and *R*- factors based on ALL data will be even larger.

### Fractional atomic coordinates and isotropic or equivalent isotropic displacement parameters (Å^2^)

**Table:**

	*x*	*y*	*z*	*U*_iso_*/*U*_eq_	
Cl1	0.0000	0.0000	0.67845 (6)	0.03585 (15)	
P1	0.0000	0.0000	0.17782 (6)	0.02407 (13)	
N1	0.08364 (13)	0.05683 (9)	0.04823 (17)	0.0305 (3)	
H1C	0.0684	0.0528	−0.0578	0.037*	
N2	−0.07306 (14)	0.06507 (10)	0.30836 (17)	0.0347 (3)	
H2A	−0.0623	0.0560	0.4146	0.042*	
C1	0.18316 (15)	0.11449 (11)	0.0998 (2)	0.0327 (4)	
H1A	0.2487	0.1016	0.0248	0.039*	
H1B	0.2067	0.0958	0.2125	0.039*	
C2	0.16072 (13)	0.21915 (10)	0.1000 (2)	0.0255 (3)	
C3	0.06933 (14)	0.25994 (12)	0.0093 (2)	0.0309 (3)	
H3A	0.0193	0.2226	−0.0548	0.037*	
C4	0.05265 (16)	0.35639 (13)	0.0142 (3)	0.0396 (4)	
H4A	−0.0090	0.3832	−0.0461	0.047*	
C5	0.12664 (17)	0.41274 (12)	0.1078 (3)	0.0435 (5)	
H5A	0.1142	0.4772	0.1120	0.052*	
C6	0.21920 (16)	0.37315 (12)	0.1953 (3)	0.0394 (4)	
H6A	0.2703	0.4111	0.2564	0.047*	
C7	0.23623 (14)	0.27655 (11)	0.1922 (2)	0.0301 (3)	
H7A	0.2984	0.2502	0.2520	0.036*	
C8	−0.15598 (16)	0.13807 (12)	0.2581 (2)	0.0342 (4)	
H8A	−0.2355	0.1160	0.2787	0.041*	
H8B	−0.1480	0.1491	0.1379	0.041*	
C9	−0.13829 (14)	0.22969 (11)	0.3506 (2)	0.0265 (3)	
C10	−0.03555 (14)	0.25145 (12)	0.4376 (2)	0.0311 (3)	
H10A	0.0248	0.2075	0.4437	0.037*	
C11	−0.02213 (15)	0.33760 (13)	0.5152 (3)	0.0392 (4)	
H11A	0.0472	0.3514	0.5725	0.047*	
C12	−0.11172 (17)	0.40365 (12)	0.5081 (3)	0.0419 (4)	
H12A	−0.1024	0.4617	0.5597	0.050*	
C13	−0.21480 (16)	0.38267 (12)	0.4238 (3)	0.0374 (4)	
H13A	−0.2752	0.4266	0.4191	0.045*	
C14	−0.22821 (14)	0.29623 (12)	0.3465 (2)	0.0317 (3)	
H14A	−0.2982	0.2824	0.2909	0.038*	

### Atomic displacement parameters (Å^2^)

**Table:**

	*U*^11^	*U*^22^	*U*^33^	*U*^12^	*U*^13^	*U*^23^
Cl1	0.0282 (3)	0.0634 (4)	0.0159 (2)	−0.0003 (3)	0.000	0.000
P1	0.0382 (3)	0.0190 (2)	0.0151 (2)	0.0001 (2)	0.000	0.000
N1	0.0447 (8)	0.0280 (6)	0.0188 (6)	−0.0127 (6)	−0.0030 (6)	−0.0009 (5)
N2	0.0570 (9)	0.0301 (6)	0.0169 (6)	0.0133 (7)	0.0002 (6)	−0.0021 (5)
C1	0.0334 (8)	0.0262 (7)	0.0387 (9)	−0.0025 (6)	−0.0071 (7)	−0.0007 (7)
C2	0.0281 (7)	0.0251 (7)	0.0234 (7)	−0.0037 (6)	0.0038 (6)	−0.0003 (6)
C3	0.0296 (7)	0.0322 (8)	0.0309 (8)	−0.0019 (6)	−0.0011 (6)	−0.0003 (7)
C4	0.0343 (8)	0.0371 (9)	0.0473 (11)	0.0062 (7)	0.0032 (8)	0.0078 (8)
C5	0.0431 (10)	0.0251 (8)	0.0624 (13)	−0.0012 (7)	0.0105 (9)	0.0014 (8)
C6	0.0394 (9)	0.0304 (8)	0.0483 (11)	−0.0120 (7)	0.0038 (9)	−0.0070 (8)
C7	0.0288 (7)	0.0311 (8)	0.0305 (8)	−0.0046 (6)	0.0020 (7)	−0.0007 (7)
C8	0.0399 (9)	0.0340 (8)	0.0288 (8)	0.0069 (7)	−0.0046 (7)	−0.0056 (7)
C9	0.0297 (7)	0.0273 (7)	0.0225 (8)	0.0029 (6)	0.0037 (6)	0.0023 (6)
C10	0.0258 (7)	0.0320 (7)	0.0354 (8)	0.0049 (6)	0.0031 (7)	−0.0015 (7)
C11	0.0277 (8)	0.0379 (9)	0.0520 (11)	−0.0061 (7)	0.0008 (8)	−0.0068 (8)
C12	0.0424 (10)	0.0238 (8)	0.0595 (12)	−0.0031 (7)	0.0106 (9)	−0.0052 (8)
C13	0.0357 (9)	0.0284 (8)	0.0481 (11)	0.0093 (7)	0.0061 (8)	0.0055 (7)
C14	0.0299 (7)	0.0330 (8)	0.0323 (9)	0.0064 (6)	−0.0019 (7)	0.0041 (7)

### Geometric parameters (Å, °)

**Table:**

P1—N1^i^	1.6166 (14)	C5—H5A	0.9300
P1—N1	1.6166 (14)	C6—C7	1.391 (2)
P1—N2^i^	1.6184 (14)	C6—H6A	0.9300
P1—N2	1.6184 (14)	C7—H7A	0.9300
N1—C1	1.456 (2)	C8—C9	1.511 (2)
N1—H1C	0.8600	C8—H8A	0.9700
N2—C8	1.459 (2)	C8—H8B	0.9700
N2—H2A	0.8600	C9—C10	1.391 (2)
C1—C2	1.514 (2)	C9—C14	1.395 (2)
C1—H1A	0.9700	C10—C11	1.382 (3)
C1—H1B	0.9700	C10—H10A	0.9300
C2—C3	1.390 (2)	C11—C12	1.388 (3)
C2—C7	1.393 (2)	C11—H11A	0.9300
C3—C4	1.389 (3)	C12—C13	1.380 (3)
C3—H3A	0.9300	C12—H12A	0.9300
C4—C5	1.379 (3)	C13—C14	1.385 (2)
C4—H4A	0.9300	C13—H13A	0.9300
C5—C6	1.380 (3)	C14—H14A	0.9300
			
N1^i^—P1—N1	101.15 (10)	C5—C6—C7	120.14 (17)
N1^i^—P1—N2^i^	114.83 (8)	C5—C6—H6A	119.9
N1—P1—N2^i^	113.07 (9)	C7—C6—H6A	119.9
N1^i^—P1—N2	113.07 (9)	C6—C7—C2	120.37 (17)
N1—P1—N2	114.83 (8)	C6—C7—H7A	119.8
N2^i^—P1—N2	100.55 (11)	C2—C7—H7A	119.8
C1—N1—P1	124.13 (12)	N2—C8—C9	113.49 (14)
C1—N1—H1C	117.9	N2—C8—H8A	108.9
P1—N1—H1C	117.9	C9—C8—H8A	108.9
C8—N2—P1	124.45 (12)	N2—C8—H8B	108.9
C8—N2—H2A	117.8	C9—C8—H8B	108.9
P1—N2—H2A	117.8	H8A—C8—H8B	107.7
N1—C1—C2	115.22 (14)	C10—C9—C14	118.34 (15)
N1—C1—H1A	108.5	C10—C9—C8	123.02 (14)
C2—C1—H1A	108.5	C14—C9—C8	118.64 (15)
N1—C1—H1B	108.5	C11—C10—C9	120.76 (15)
C2—C1—H1B	108.5	C11—C10—H10A	119.6
H1A—C1—H1B	107.5	C9—C10—H10A	119.6
C3—C2—C7	119.02 (14)	C10—C11—C12	120.27 (17)
C3—C2—C1	122.52 (14)	C10—C11—H11A	119.9
C7—C2—C1	118.45 (15)	C12—C11—H11A	119.9
C4—C3—C2	120.11 (16)	C13—C12—C11	119.65 (16)
C4—C3—H3A	119.9	C13—C12—H12A	120.2
C2—C3—H3A	119.9	C11—C12—H12A	120.2
C5—C4—C3	120.59 (17)	C12—C13—C14	120.01 (16)
C5—C4—H4A	119.7	C12—C13—H13A	120.0
C3—C4—H4A	119.7	C14—C13—H13A	120.0
C4—C5—C6	119.74 (16)	C13—C14—C9	120.96 (16)
C4—C5—H5A	120.1	C13—C14—H14A	119.5
C6—C5—H5A	120.1	C9—C14—H14A	119.5
			
N1^i^—P1—N1—C1	−174.86 (17)	C5—C6—C7—C2	0.5 (3)
N2^i^—P1—N1—C1	−51.55 (15)	C3—C2—C7—C6	1.0 (2)
N2—P1—N1—C1	63.05 (15)	C1—C2—C7—C6	179.71 (17)
N1^i^—P1—N2—C8	−56.69 (16)	P1—N2—C8—C9	−132.58 (14)
N1—P1—N2—C8	58.69 (17)	N2—C8—C9—C10	17.1 (2)
N2^i^—P1—N2—C8	−179.61 (19)	N2—C8—C9—C14	−163.97 (15)
P1—N1—C1—C2	−101.97 (17)	C14—C9—C10—C11	−1.2 (3)
N1—C1—C2—C3	−19.8 (2)	C8—C9—C10—C11	177.70 (17)
N1—C1—C2—C7	161.45 (15)	C9—C10—C11—C12	0.4 (3)
C7—C2—C3—C4	−1.4 (2)	C10—C11—C12—C13	0.4 (3)
C1—C2—C3—C4	179.90 (17)	C11—C12—C13—C14	−0.3 (3)
C2—C3—C4—C5	0.4 (3)	C12—C13—C14—C9	−0.6 (3)
C3—C4—C5—C6	1.0 (3)	C10—C9—C14—C13	1.4 (2)
C4—C5—C6—C7	−1.5 (3)	C8—C9—C14—C13	−177.61 (16)

Symmetry codes: (i) −*x*, −*y*, *z*.

### Hydrogen-bond geometry (Å, °)

**Table:**

*D*—H···*A*	*D*—H	H···*A*	*D*···*A*	*D*—H···*A*
N1—H1C···Cl1^ii^	0.86	2.35	3.1848 (17)	163
N2—H2A···Cl1	0.86	2.35	3.1855 (18)	165

Symmetry codes: (ii) *x*, *y*, *z*−1.

## References

[bb1] Bickley, J. F., Copsey, M. C., Jeffery, J. C., Leedham, A. P., Russell, C. A., Stalke, D., Steiner, A., Stey, T. & Zacchini, S. (2004). *Dalton Trans.* pp. 989–995. 10.1039/b400798k 15252677

[bb2] Flack, H. D. (1983). *Acta Cryst.* A**39**, 876–881.

[bb3] Hart, W. A. & Sisler, H. H. (1964). *Inorg. Chem.* **3**, 617–622.

[bb4] Horstmann, S. & Schnick, W. (1996). *Z. Naturforsch. Teil B*, **51**, 127–132.

[bb5] Levason, W., Reid, G. & Webster, M. (2006). *Acta Cryst.* C**62**, o438–o440. 10.1107/S0108270106015915 16823225

[bb6] Macrae, C. F., Bruno, I. J., Chisholm, J. A., Edgington, P. R., McCabe, P., Pidcock, E., Rodriguez-Monge, L., Taylor, R., van de Streek, J. & Wood, P. A. (2008). *J. Appl. Cryst.* **41**, 466–470.

[bb7] Schiemenz, B., Wessel, T. & Pfirmann, R. (2003). US Patent No. 6 645 904.

[bb8] Sheldrick, G. M. (2008). *Acta Cryst.* A**64**, 112–122. 10.1107/S0108767307043930 18156677

[bb9] Siemens (1989). *P3/PC* Siemens Analytical X-ray Instruments Inc., Madison, Wisconsin, USA.

